# Comparison of deep and moderate neuromuscular blockade in microwave ablation of liver tumours: a randomized-controlled clinical trial

**DOI:** 10.1038/s41598-021-81913-1

**Published:** 2021-01-27

**Authors:** Pui San Loh, Chai Hong Yeong, Naeema S. Masohood, Norshazriman Sulaiman, Rafdzah Ahmad Zaki, Kamil Fabell, Basri Johan Jeet Abdullah

**Affiliations:** 1 Department of Anaesthesiology, Faculty of Medicine, University of Malaya, 50603 Kuala Lumpur, Malaysia; 2 School of Medicine, Faculty of Health and Medical Sciences, Taylor’s University, 47500 Subang Jaya, Selangor Malaysia; 3 Department of Biomedical Imaging, Faculty of Medicine, University of Malaya, 50603 Kuala Lumpur, Malaysia; 4 Department of Social and Preventive Medicine, Faculty of Medicine, University of Malaya, 50603 Kuala Lumpur, Malaysia

**Keywords:** Gastroenterology, Medical research, Oncology

## Abstract

Microwave ablation (MWA) is gaining popularity for the treatment of small primary hepatocellular carcinoma and metastatic lesions especially if patients are not candidates for surgical resection. Deep neuromuscular blockade (DMB) is perceived to improve surgical working conditions compared to moderate neuromuscular blockade (MMB) but no studies have examined the same benefits in MWA of liver tumours. This study aimed to compare the clinical outcomes of DMB and MMB in MWA of liver tumours in terms of liver excursion, performance scores by the interventional radiologists and patients, requirements of additional muscle relaxants and complications. 50 patients were recruited and 45 patients (22 in MMB group, 23 in DMB group) completed the study. The mean liver excursion for the MMB group (1.42 ± 1.83 mm) was significantly higher than the DMB group (0.26 ± 0.38 mm) (p = 0.001). The mean Leiden-Surgical Rating Scale (L-SRS) rated by the two interventional radiologists were 4.5 ± 0.59 and 3.6 ± 0.85 for the DMB and MMB groups, respectively (p = 0.01). There was also statistically significant difference on patient satisfaction scores (0–10: Extremely Dissatisfied–Extremely Satisfied) between DMB (8.74 ± 1.1) and MMB (7.86 ± 1.25) groups (p = 0.01). 5 patients from MMB group and none from DMB group required bolus relaxant during the MWA procedure. Adverse events were also noted to be more severe in the MMB group. In conclusion, DMB significantly reduced liver excursion and movement leading to improved accuracy, safety and success in ablating liver tumour.

## Introduction

In most hospitals, there is an increasing demand to provide anaesthesia for patients undergoing procedures outside the operating theatre. Technological advances have expanded the demand for complex radiological procedures and minimally invasive interventions. Percutaneous thermal ablation is one of the main treatment options for small solid tumours of hepatocellular carcinoma (HCC) and liver metastases. It is a minimally invasive technique that delivers heat (between 60 and 90 °C) to the tumour under imaging guidance to induce coagulation necrosis^1^. The most commonly used methods of thermal ablation for liver tumour are radiofrequency ablation (RFA) and microwave ablation (MWA), of which in recent years, MWA has been gaining momentum over RFA due to several advantages including ability to treat larger and multiple lesions at one setting^2^.

Movement during image-guided thermal ablation may reduce the accuracy of tumour targeting and increase the risk of complications. Large hepatic motion during breathing also causes technical difficulties for the interventional radiologist to perform the procedure safely^3^. Holland et al.^4^ investigated diaphragm and cardiac motion during breath-holding in spontaneously breathing patients and found that during a breath-hold, the diaphragm moved upward. At end expiration, the velocity of the diaphragm during suspended breathing was constant (average 0.15 mm/s). At end inspiration, motion of the diaphragm during suspended breathing was more complex (ranged between 0.1 and 7.9 mm/s). During a 20 s breath-hold, mean displacement of the diaphragm was 25% of that during normal breathing. This is a significant range of movement, which could translate into inaccuracies in needle placement, incomplete ablation and insufficient ablative safety margin.

Anaesthetic options for thermal ablation of liver tumour include local anaesthesia with sedation or general anaesthesia (GA). Treatments done under local anaesthesia require patients’ cooperation for breath-holding as it is critical for accurate needle placement. Therefore, common patient complains are significant pain during procedure leading to inability to tolerate or complete the procedure. GA provides a better option for analgesia and tolerance of procedure^5^. Respiration-related liver motion can also be minimized by anaesthesia and neuromuscular blockade. A retrospective study^6^ found that treatment of small HCC by RFA under GA was associated with reduced risk of cancer recurrence. However, no effects of anaesthetic technique on overall survival have been reported.

Recent studies in laparoscopic surgeries have shown that GA with deep neuromuscular blockade (DMB) improves surgical conditions. Staehr-Rye et al.^7^ found that DMB marginally improved surgical conditions during laparoscopic cholecystectomies. Martini et al.^8^ reported similar results whereby surgeons rated better operating conditions in the DMB group (p < 0.001) in laparoscopic prostatectomy or nephrectomy. Blobner et al.^9^ studied patients in laparoscopic cystectomy and found that better operating conditions occur in the “deep block” group with more events associated with lack of muscle relaxation in the “no block” group. Similar results were also reported by Dubois et al.^10^ of worse operating conditions in the “shallow block” group in laparoscopic hysterectomies. On the other hand, Honing et al.^11^ recently reported that DMB did not improve surgical conditions over moderate neuromuscular blockade (MMB) in patients receiving sevoflurane anaesthesia for laparoscopic renal surgery. All of these studies utilized surgeon rating scale on a numerical or visual analogue scale as a tool to measure optimum operating conditions.

For thermal ablation, adequate depth of neuromuscular blockade is paramount, as the loss of muscle paralysis would pose technical difficulties for needle placement and insufficient ablative safety margin^8^. The introduction of sugammadex into the armamentarium of anaesthesia has enabled anaesthetists to allow deeper levels of neuromuscular blockade with rocuronium for various indications^12^. As the dose of sugammadex can be tailored according to the level of block and with its known predictability of reversal, sugammadex makes it safe to reverse patients even at very deep levels of neuromuscular block^13^.

Hypothetically, DMB would provide better conditions for percutaneous thermal ablation procedures. The main objective of this study was to compare the different depths of neuromuscular block between DMB and MMB groups during MWA for primary or secondary hepatic tumours to look at the differences in mean liver excursions as the primary outcome. The ease of performing the MWA, patient satisfaction and associated complications represents the secondary outcomes of the study.

## Methods

### Study design

This was a single-centre, prospective, double-blinded, randomized controlled clinical trial involving patients undergoing computed tomography (CT)-guided MWA of primary and secondary liver tumours at the University of Malaya Medical Centre. The study protocol was approved by the Medical Ethics Committee of the University of Malaya Medical Centre (MECID No. 20151-930). The clinical trial was registered in the Australian New Zealand Clinical Trials Registry (Clinical Trial Registration Number: ACTRN12615000838516, Date registered: 11/08/2015). The study was performed in accordance with the principles of the Declaration of Helsinki, and written informed consent was obtained from all participants prior to enrolment.

### Patients selection and randomization

Patients with confirmed diagnosis of primary or secondary liver tumour and referred for MWA by the primary team were recruited. Patients who had any contraindication to MWA and GA, allergy to any drugs used in the study such as rocuronium, sugammadex, iodinated contrast media, pregnant or breastfeeding and patients who were unable or refuse to give informed consent were excluded from the study. Number of patients required to achieve at least 80% power (1 − β) at α-level 5% was 22 patients in each group, as calculated using the open source software (OpenEpi version 3.03a) based on estimation of difference in mean liver excursion (and standard deviation, SD) of 1.0 mm and difference in mean performance score of 1.0 between the two groups. After accounting for 10% dropout, a sample size of 50 patients was determined.

Computer-generated randomization was done using the Research Randomizer software. Subjects were allocated a patient index number from 1 to 50 and randomly assigned to MMB (Group 1) or DMB (Group 2) at the start of the study. The allocated group (1 or 2) was documented with patient’s index number and made known to the attending anaesthetist when the subjects corresponding to the sequence of index number were recruited. The patient, the interventional radiologist and the research team were blinded to the allocated groups. Only the attending anaesthetists knew the allocated groups to enable administration of drugs, monitoring and management.

### Conduct of anaesthesia

All patients were admitted one day prior to procedure for pre-operative assessment and informed consent. The patients were then fasted for at least 6 h prior to the procedure. During the intervention, the attending anaesthetic team would prepare the necessary drugs as per protocol and allocated group (either MMB or DMB). The lead anaesthetist was responsible for conduct of anaesthesia, administration of the muscle relaxant and level of neuromuscular block whilst patient and interventional radiologist remained blinded.

Patient had routine monitoring with ECG, pulse oximetry, non-invasive blood pressure. The depth of neuromuscular blockade was monitored with a peripheral nerve stimulator (TOF-Watch, Organon, Ireland) at the flexor pollicis longus for ease of access. After pre-oxygenation, patients were induced with intravenous (IV) fentanyl 1–2 mcg/kg and IV propofol 2 mg/kg. Neuromuscular monitoring was calibrated before administering muscle relaxant and intubated at train-of-four (TOF) count 0. Anaesthesia was maintained with oxygen, air and sevoflurane at minimum alveolar concentration (MAC) value of 1.0–1.3.

In the DMB group, patients received 1.0 mg/kg of IV rocuronium bolus at induction followed by infusion at the rate of 0.6 mg/kg/h to maintain post-tetanic count (PTC) of 1–2 or adjusted accordingly by increments of 0.1 mg/kg/h to maintain PTC 1–2. Patients in the MMB group would receive 0.5 mg/kg IV atracurium bolus followed by infusion at 0.3 mg/kg/h to maintain TOF of 1 or 2 or adjusted accordingly by increments of 0.1 mg/kg/h to maintain TOF 1–2. Boluses of muscle relaxant as top-up could be requested by the interventional radiologist and documented.

Patients were ventilated with pressure control to achieve a tidal volume of not more than 0.7 ml/kg and end-tidal carbon dioxide (ETCO_2_) in the range of 35–40 mmHg. During the procedure, ventilation was intermittently suspended at varying lengths of time as required by the radiologist and immediately resumed if the oxygen saturation dropped below 96% or > 4% from baseline. During suspended ventilation, passive oxygenation was maintained with oxygen flow of 2 l/min with the adjustable pressure-limiting (APL) valve turned to minimal.

All patients received 0.1 mg/kg IV morphine and 4 mg IV dexamethasone after induction. Further boluses of analgesia were given after extubation based on pain scores in recovery. Hypotensive episodes post induction (attributed to vasodilatory effects of anaesthetic agents) was treated with IV crystalloids/colloids and small boluses of 6 mg IV ephedrine or 0.1 mg IV phenylephrine, if needed.

### Microwave ablation

Two interventional radiologists were involved in this study. Standard surgical preparation and draping were performed prior to MWA. Using computed tomography (CT) guidance, the tumour was localized and the optimal ablative approach was determined by the interventional radiologist. A MWA needle was then inserted percutaneously into the tumour under aseptic technique and connected to a microwave generator (KY-2000, Canyon, China) via a coaxial cable. The electromagnetic microwave was emitted from the exposed, non-insulated portion of the needle from a generator capable of producing 60 W of power at a frequency of 915 MHz. Each ablation took about 10 min per 3 cm lesion.

### Analysis of liver excursion

A five-phase CT of the abdomen was done immediately after the MWA procedure to assess the performance of the ablation. To obtain the mean liver excursion, the differences in the liver position (taken at the level of the diaphragm) among the different phases of the CT scan were obtained. This translated into the cranio-caudal movement (in mm) of the liver. The measurements were taken between the early arterial, late arterial and portal venous phase of the CT.

### Post-procedural management

After completion of the MWA procedure, patients in DMB group were reversed with 4 mg/kg IV sugammadex, while patients in MMB group had 0.05 mg/kg IV neostigmine and 0.02 mg/kg IV atropine. Patients were extubated when TOF ratio became 0.9 or more. After extubation, patients’ vital signs were monitored in the post-anaesthetic recovery area. If the attending anaesthetist decided that the patient was not suitable for extubation, then plans to continue ventilation in Intensive Care Unit (ICU) would be arranged and the same data for the first half an hour in ICU were recorded. All adverse events were divided to procedure-related or non-procedure related. Their time of occurrence, treatment and outcomes during the study were recorded with their severity classified according to the National Cancer Institute Common Terminology Criteria for Adverse Events Version 4.0.

### Performance evaluation by interventional radiologists and patients

Performance level of the overall procedure was then rated by the interventional radiologists using a 5-point Leiden-Surgical Rating Scale (L-SRS) (Table 1) upon completion of the procedure. The L-SRS rating system has been validated by multiple researchers previously^8,14,15^, and the interventional radiologists involved in this study were familiar with the system. Patients were also asked to rate their satisfaction level about the overall anaesthetic and MWA procedure using a 11-point Likert scale (0–10: Extremely Dissatisfied–Extremely Satisfied) within 24 h after procedure.

**Table 1:** The L-SRS rating scale^15^.

Scale		Description
1	Extremely poor	The interventional radiologist is unable to work because of coughing or because of the inability to obtain a visible treatment field
2	Poor	There is a visible treatment field, but the interventional radiologist is severely hampered by inadequate muscle relaxation with continuous muscle contractions, or both with the hazard of tissue damage
3	Acceptable	There is a wide visible treatment field, but muscle contractions, movements, or both occur regularly causing some interference with the interventional radiologist’s work
4	Good	There is a wide treatment working field with sporadic muscle contractions, movements or both
5	Optimal	There is a wide treatment working field without any movement or contractions

### Statistical analysis

Statistical analysis was done using SPSS software (SPSS Statistics version 24.0, IBM, USA). General characteristics of the study samples were analysed by descriptive statistics and cross-tabulation where applicable. Performance scores by the interventional radiologists and patient satisfaction scores as well as differences in the mean liver excursion in post-procedure CT were analysed using independent sample t-tests to compare means between the studied groups. Values are presented as means ± standard deviation (SD), unless otherwise stated. A p-value < 0.05 was considered as statistically significant different.

## Results

### Patients demography

50 patients were recruited in this study, however 5 patients did not complete the post-ablation CT scan and hence were excluded from the analysis. Out of the 45 patients (30 males, 15 females), 22 patients were in the MMB group (48.9%) while 23 patients were in the DMB group (51.1%). Demography of the patients are tabulated in Table 2. The physical status of the patients was classified to Class I (normal healthy patient), II (patient with mild systemic disease) and III (patient with severe systemic disease) according to the American Society of Anesthesiologists (ASA) Physical Status Classification System. No statistically significant difference was found in any of the patient characteristics.

**Table 2:** Patients demography and characteristics (n = 45).

	MMB (n = 22)	DMB (n = 23)	p-value
Age (years)	63.0 ± 6.7	61.5 ± 12.5	0.60
Sex (M/F)	16/6	14/9	0.40
Weight (kg)	65.1 ± 11.3	63.4 ± 16.4	0.70
Height (cm)	165.8 ± 11.3	162.8 ± 7.9	0.30
BMI (kg/m^2^)	23.7 ± 4.0	23.8 ± 5.7	0.90
ASA physical status classification (I/II/III)	1/21/0	6/16/1	0.10

*M* male, *F* female, *BMI* Body Mass Index, *ASA* American Society of Anesthesiologists.

There were 33 (73.3%) patients with primary HCC and 12 (26.7%) with secondary tumours. No statistically significant difference was found in the distribution of primary or secondary tumour between the DMB and MMB groups. Of the primary HCC, four patients (12.1%) had recurrent tumour with history of liver ablation treatment. Analysis of the aetiology of the primary HCC found 14 patients (42.4%) with underlying Hepatitis B, 7 patients (21.2%) with Hepatitis C, 3 patients (9.1%) with cryptogenic HCC and remaining 9 patients (27.3%) with unspecified cause of HCC. Of the 12 patients with secondary liver tumours, 3 had colorectal carcinoma (25.0%), 2 had breast carcinoma (16.7%) while the remainder had lung carcinoma, pancreatic carcinoma, nasopharyngeal carcinoma, cholangiocarcinoma, ureteric carcinoma, oesophageal carcinoma and carcinoid tumour (1 patient each, 8.3%). Table 3 shows the number of lesions per patient according to the depth of neuromuscular blocks. There was more single lesion in the MMB group and 2 to 5 lesions in the DMB group (p = 0.04). The size of lesions ranged between 0.3 and 6.9 cm (mean 2.46 ± 0.99 cm) with no significant difference in lesion size between groups (p > 0.05).

**Table 3:** Number of lesions by depth of neuromuscular block.

Number of lesions	Depth of neuromuscular block, count (%)		Total (n = 45)
	MMB (n = 22)	DMB (n = 23)	
1	15 (33.3)	9 (20.0)	24 (53.3)
2 to 5	7 (15.6)	13 (28.9)	20 (44.4)
8	0 (0.0)	1 (2.2)	1 (2.2)

### Intraoperative data analysis

At induction, all patients received 99.4 ± 3.7 mcg of IV fentanyl and 114 ± 30.9 mg of IV propofol. 3.3 ± 1.5 IV morphine was given as analgesia. The haemodynamic parameters at induction and during procedure are summarized in Table 4. In the MMB group, the mean dose of IV atracurium given at induction was 26.6 ± 6.7 mg and the mean total dose used was 42.5 ± 13.6 mg. As for rocuronium the mean dose of IV rocuronium given to the DMB group at induction was 46.9 ± 13.2 mg and the total dose used was 78.5 ± 20.6 mg.

**Table 4:** Mean haemodynamic parameters of study samples.

	MMB		DMB	
	At induction	During procedure	At induction	During procedure
SBP (mmHg)	147.1 ± 19.5	97.8 ± 18.8	134.5 ± 21.5	103.5 ± 19.1
DBP (mmHg)	83.7 ± 12.4	54.6 ± 8.8	75 ± 12.3	56.4 ± 14.7
HR (bpm)	78 ± 13	69 ± 12	81 ± 16	76 ± 18
SpO2 (%)	99.9 ± 0.2	99.9 ± 0.3	99.9 ± 0.4	99.9 ± 0.3

*SBP* systolic blood pressure; *DBP* diastolic blood pressure, *HR* heart rate, *SpO2* peripheral capillary oxygen saturation.

During MWA, the radiologist requested for bolus relaxant for 5 out of 22 patients in the MMB group. In contrast, no bolus relaxant was requested for all the patients in the DMB group (p = 0.02). Thus, there was a statistically significant association between depth of muscle block and request of bolus relaxant by the radiologist demonstrating that a significant number of patients in the MMB group had inadequate depth of muscle relaxation which was apparent to the radiologist during the ablation procedure. The duration of MWA was proportional to the number of lesions treated with a mean duration of 114.8 ± 55.2 min (ranged 40–250 min, p = 0.55). All patients were extubated post procedure. The mean duration from reversal to extubation was 7.5 ± 4.4 min for the MMB group, and 8.0 ± 3.4 min for the DMB group (p = 0.60).

### Liver excursion during MWA

There was statistically significant difference in liver excursions between the MMB and DMB groups (p = 0.001). The mean liver excursion was higher in the MMB group (1.42 ± 1.83 mm) than the DMB group (0.26 ± 0.38 mm) with a wider range of movement with MMB (0–8 mm) compared to DMB (0–1.3 mm).

### Performance scores by interventional radiologists and patients

**Figure 1:**
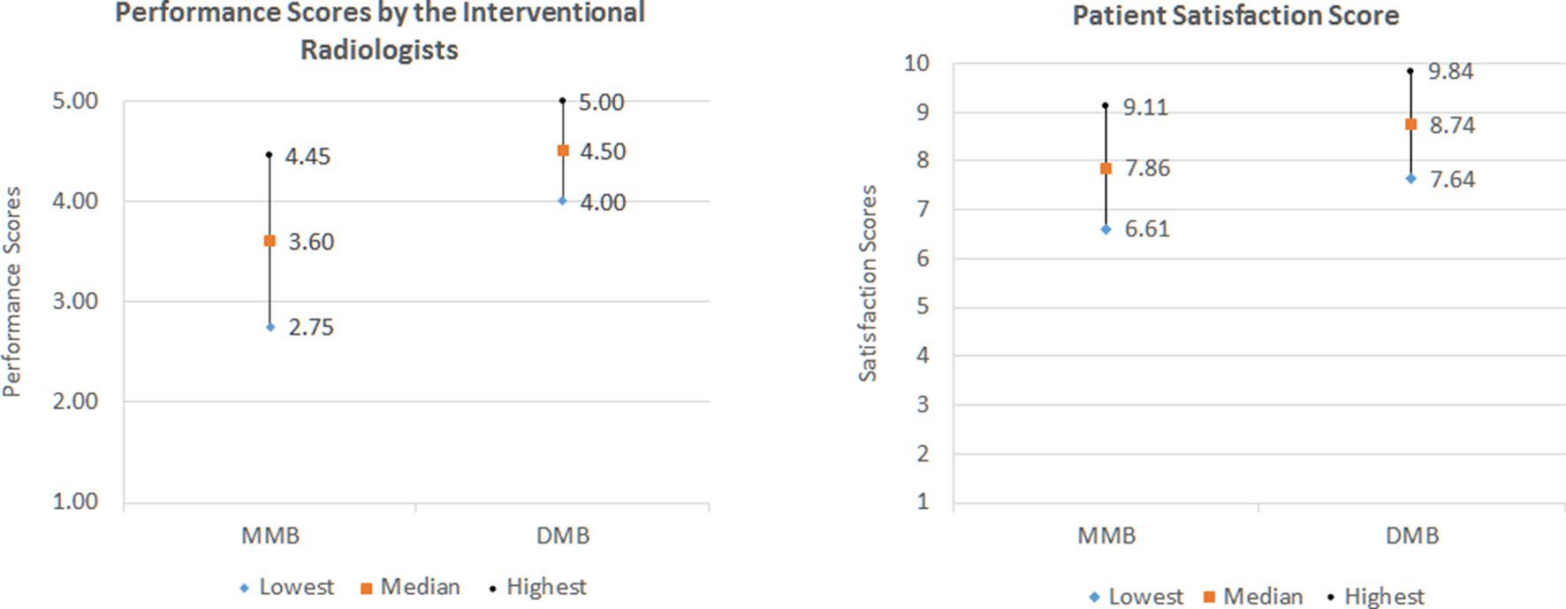
Performance scores by radiologists and patient satisfaction scores.

Performance scores by the radiologists were significantly higher in the DMB group (mean score 4.50 ± 0.59) compared to the MMB group (mean score 3.60 ± 0.85) (p = 0.001). There was also statistically significant difference on patient satisfaction scores between DMB (8.74 ± 1.1) and MMB (7.86 ± 1.25) groups (p = 0.01). Figure 1 shows the results of the performance and patient satisfaction scores rated by the interventional radiologists and patients, respectively.

### Adverse events

Out of 45 patients, 40 patients (88.9%) had no pain or minimal pain during recovery and did not require rescue analgesics. Only 5 patients (11.1%) had significant pain requiring rescue analgesics in the recovery phase with no significant difference (p = 0.30) between the pain scores of MMB and DMB groups. Most patients were fully awake, comfortable with no nausea or vomiting (41 patients, 91.1%). Only 3 had nausea alone (6.7%) and 1 patient with severe nausea and vomiting (2%). There were no incidences of respiratory compromise with desaturation due to poor reversal.

Overall, 7 patients (15.6%) developed procedure-related adverse events. Out of these, 85.7% developed during the MWA procedure. The most common adverse event was subcapsular bleeding that occurred in 5 patients (11.1%). Two of the patients from the MMB group had severe subcapsular bleeding (Grade 3) with haemodynamic compromise requiring immediate embolization of the hepatic artery and 2-day stay in ICU. The other 3 in DMB who also developed subcapsular bleed (Grade 1) only required conservative management. There was one case of bleeding from the puncture site (Grade 2) during procedure that resolved with platelet transfusion. Another patient who had prior bilateral pleural effusion from the CT guided image required bilateral pigtail drain insertion immediately after the procedure and was not considered as a procedure-related complication. Majority of the patients were discharged within 3 days post procedure (98%). Only two patients had prolonged stay for 5 days after subcapsular bleed which required close observation in the ICU for 2 days.

## Discussion

Thermal ablation procedures like MWA require complete immobilization throughout the procedure for accurate needle placement, which theoretically translates into good ablation volume with adequate safety margin. Patient immobility is therefore essential especially when the lesions are located near vital structures such as the artery or vein, diaphragm, lung parenchyma and bowels. The standard anaesthetic care for MWA of liver tumours patients in our centre is GA with MMB and intermittent positive pressure ventilation. This was the first study done to compare the performance of DMB and MMB during MWA of hepatic tumours. The results demonstrate significant difference in liver excursion, performance scores and post-procedure patient satisfaction.

Percutaneous thermal ablation of primary HCC and secondary hepatic metastases have slightly higher recurrence rate compared to primary surgical resection^16^. Thus, it is imperative that the ablation volume and safety margin are achieved accurately as this may affect the patients’ long-term survival. This study showed that DMB reduced mean liver excursion significantly compared to MMB and able to achieve sub-millimetre margins (0.26 ± 0.38 mm). Sufficient ablation margin is the most significant predictor of local tumour progression free survival (LTPFS) in MWA. A minimum safety margin of 5 mm is mandatory to achieve complete tumour necrosis^17^. By minimizing liver excursion during the procedure, a good ablative and safety margin can be achieved with the aim to reduce recurrence and improve overall survival rates. Therefore, anaesthesia plays a key role in the overall success rate of percutaneous MWA. Currently there is no consensus on the best anaesthetic approach to patients undergoing CT-guided MWA or RFA. The anaesthetic practice and prescriptions could vary widely, ranging from local anaesthesia alone, to monitored anaesthesia care, to regional anaesthesia, to combined regional and GA^18^. To our knowledge, this is the first study comparing DMB and MMB performance outcomes in CT-guided percutaneous MWA of liver tumour.

In this study, DMB was achieved with rocuronium to enable safe reversal with sugammadex. On the other hand, MMB was achieved with atracurium as per routine institutional practice and reversal with neostigmine. The different drugs should not affect the outcome of this study, as the end point is the difference in the depth of muscle relaxation. DMB was set at PTC 1 to 2, while MMB was set at TOF 1 to 2, monitored with peripheral nerve stimulator (PNS) similar to prior studies comparing depths of muscle relaxation^8^. During the study, apparent signs of diaphragmatic movement were noted by the radiologist in the MMB group. This movement hindered the procedure and hence, additional muscle relaxant was requested and yet, the TOF at the flexor pollicis was still 1 to 2 for MMB. This effect was not seen in the DMB group. This phenomenon is explained by the different sensitivities of the diaphragm, abdominal muscle, and peripheral muscles to non-depolarizing neuromuscular blocking drugs. Other possible mechanisms could be due to variations in acetylcholine receptor density, acetylcholine release, acetylcholinesterase activity, fibre composition, blood flow and muscle temperature^19^.

Previous studies comparing depths of muscle relaxation during anaesthesia were only done in laparoscopic surgeries and they looked at the surgical working conditions^20^. All of these studies observed positive results in favour of DMB as it improved surgical working conditions as assessed by L-SRS rating system^21^. The present study also utilized a 5-point L-SRS rating system to grade the performance of the MWA procedure by the interventional radiologists. The DMB group attained higher performance score as patients in this group were relatively still especially during suspended ventilation when needle insertion was performed. As explained earlier, at most times, diaphragmatic movements were obvious in the MMB group and the interventional radiologist would request for bolus relaxant to be given. Therefore, although there were more lesions per procedure in the DMB group, the performance scores given were still significantly higher than for the MMB group.

This study also showed equal procedure-related complication rates between DMB and MMB groups. However, the adverse events in the MMB group were severe and required additional invasive treatment, ICU care and extra days of hospitalization. In terms of the effects of neuromuscular blockade and reversal, with the use of sugammadex in the DMB group and neostigmine in MMB, there was no incidence of residual paralysis nor adverse respiratory complications with the guidance of neuromuscular monitoring.

However, limitations apply to the interpretation of these results. Other clinical outcomes such as tumour recurrence, the need for a repeat tumour ablation and long-term survival rates need to be evaluated. Moreover, this is a single centre study where else, a pragmatic multicentre randomised controlled trial that include a larger sample size will be needed to validate these findings.

## Conclusion

This study is among the few published to compare the performance of DMB and MMB for MWA of hepatic tumours. Results showed that DMB significantly reduced liver excursion and also yielded significantly higher performance scores by both interventional radiologists and patients. There is also no perceived diaphragmatic movement by the radiologists that required additional boluses of muscle relaxant, hence improving performance for better accuracy and success in ablating a safe margin. Lastly, adverse events were noted to be more severe in the MMB group. However, further studies are needed to explore patients’ outcome in terms of long-term adverse effects and tumour recurrence rate.
